# Supplementary material for: Mechanisms governing the pioneering and redistribution capabilities of the non-classical pioneer PU.1
Source: Nat Commun. 2020 Jan 21;11:402. doi: 10.1038/s41467-019-13960-2 (PMC6972792; doi:10.1038/s41467-019-13960-2)
Supplement: Supplementary file 7 — Source data [file 41467_2019_13960_MOESM7_ESM.zip › Source_Data/FigureS3/FigureS3H_MotifScanOutput/homerResults/motif19.similar.html]

motif19

## Information for motif19

A
T
G
C
A
G
T
C
A
C
G
T
A
G
C
T
C
G
A
T
T
A
C
G
G
T
C
A
C
G
A
T
  
Reverse Opposite:  

G
C
T
A
C
A
G
T
A
T
G
C
G
C
T
A
T
C
G
A
T
G
C
A
T
C
A
G
T
A
C
G
  

|  |  |
| --- | --- |
| p-value: | 1e-43 |
| log p-value: | -1.006e+02 |
| Information Content per bp: | 1.640 |
| Number of Target Sequences with motif | 978.0 |
| Percentage of Target Sequences with motif | 25.30% |
| Number of Background Sequences with motif | 7209.5 |
| Percentage of Background Sequences with motif | 16.09% |
| Average Position of motif in Targets | 291.3 +/- 270.8bp |
| Average Position of motif in Background | 209.7 +/- 138.0bp |
| Strand Bias (log2 ratio + to - strand density) | 0.2 |
| Multiplicity (# of sites on avg that occur together) | 1.17 |
| Motif File: | file (matrix) reverse opposite |

### Similar de novo motifs found

|  |  |  |  |  |  |  |  |
| --- | --- | --- | --- | --- | --- | --- | --- |
| Rank | Match Score | Redundant Motif | P-value | log P-value | % of Targets | % of Background | Motif file |
| 1 | 0.966 | C G T A A C G T A G T C C G T A C G T A G T C A A T C G | 1e-42 | -97.872794 | 29.54% | 19.82% | motif file (matrix) |
| 2 | 0.770 | A G T C G A C T A C G T C G A T T A C G G T C A C A G T T G A C G C A T G C T A G T C A | 1e-25 | -58.947751 | 18.80% | 12.54% | motif file (matrix) |
